# Sexual HIV risk behaviour and associated factors among pregnant women in Mpumalanga, South Africa

**DOI:** 10.1186/1471-2393-13-57

**Published:** 2013-03-04

**Authors:** Karl Peltzer, Gladys Mlambo

**Affiliations:** 1 HIV/AIDS/SIT/and TB (HAST), Human Sciences Research Council, Pretoria, South Africa; 2 Department of Psychology, University of the Free State, Bloemfontein, South Africa; 3 ASEAN Institute for Health Development, Mahidol University, Bangkok, Thailand

## Abstract

**Background:**

The HIV risk increases during pregnancy. The elevated risk of HIV acquisition in pregnant women may be explained by behavioural and other factors. The aim of this study was to assess sexual HIV risk behaviour and its associated factors among pregnant women in Mpumalanga, South Africa.

**Methods:**

A cross-sectional study was conducted among 1 502 pregnant women (age range 18–47 years, mean age 26.6 years, standard deviation (SD) 6.1, and the mean gestational age was 6.5 months (SD 1.6). Antenatal women were selected, using systematic sampling from 63 primary care clinics and community health centres in Nkangala District. Data were collected by using a structured questionnaire and multivariate logistic regression analysis was used.

**Results:**

The majority (63%) of the participants had never used a condom with their primary sexual partner in the past 3 months, 60% were not aware of the HIV status of their sexual partner, 7.6% had a casual sexual partner in the past 3 months, 20% had two or more sexual partners in the past 12 months and 17.3% reported to have been diagnosed with a sexually transmitted infection (STI) (other than HIV) in the past 12 months. The various HIV risk behaviours were predicted, by being single and alcohol use for multiple sexual partners; by fewer antenatal visits, being HIV negative and not having used alcohol for lack of condom use; by being HIV positive, having experienced physical partner violence and psychological distress for having been diagnosed with a sexually transmitted infection (other than HIV); and by lower education, unplanned pregnancy, non-antenatal care attendance by expectant father, the belief that antiretrovirals can cure HIV and being HIV positive for having a partner with HIV positve or unknown status.

**Conclusion:**

High levels of sexual HIV risk behaviour were found during pregnancy. Pregnant women need to be informed of their increased risk of HIV and the importance of sexual HIV risk reduction including the use of condoms throughout pregnancy.

## Background

Although male to female transmission of HIV has been estimated as 2.3 times greater than female to male transmission [1,2], the risk of HIV acquisition rises for both men and women during pregnancy [3,4]. In South Africa, it is estimated that the proportion of mother-to-child transmission (MTCT) from mothers who seroconverted after their first antenatal visit was 26% in 2008 [5]. This change is likely to be due to both sexual risk behaviours and to hormonal changes affecting the genital tract mucosa or immune responses [3,6]. In fact, previous studies found an increase of HIV-1 incidence among pregnant women when compared with non-pregnant women [3].

Few studies have investigated the sexual HIV risk behavior during pregnancy. In a study conducted in Tanzania, HIV-1 risk factors were similar between pregnant and all women in the general population [7]. In Cape Town, South Africa, pregnant women reported to have had fewer male partners compared to non-pregnant women in the previous 6 months, but they also reported less condom use and were less likely to request that their partners use condoms [8]. Similarly, in Rakai, Uganda, and Cape Town, South Africa condom use were found to be less consistent during pregnancy [6,9]. In fact, among couples that associate condom use with contraception rather than risk reduction, sex may be more likely to be unprotected throughout pregnancy, greatly increasing the risk for HIV transmission, with women engaging in more HIV risk behavior overall [10]. Finally, among HIV serodiscordant or concordant couples, the desire to conceive may lead to risky behaviour despite knowledge of serostatus [11]. In Cape Town, over 30% of HIV positive women and 65% of HIV negative men attending public sector clinics reported an interest in having additional children, and in Johannesburg, 60% of HIV positive women had planned to conceive in the next year, while in both groups, most had never had a conversation with a health care worker on this issue [12]. Peltzer et al. [13] found high levels of HIV risk behaviour among couples during pregnancy in Mpumalanga, South Africa. Individuals may tend to overestimate their spouse’s, as well as their own risk of having HIV [14]. Factors associated with sexual HIV risk behaviour among pregnant women may include HIV status, partner’s HIV status [13], intimate partner violence [15,16], general relational factors (attachment anxiety) [17] and power imbalances [18]. While marital infidelity is the most important correlate of overestimation of individual and spousal HIV risk, during pregnancy, concerns regarding partners’ HIV status may be superseded by the reduced need for contraception [13].

The aim of this study was to assess sexual HIV risk behaviour and its associated factors among pregnant women in Mpumalanga, South Africa.

## Methods

### Sample and procedure

A cross-sectional study was conducted among 1 502 pregnant women, age range 18–47 years, mean age 26.6 years, standard deviation (SD) 6.1; 98% were black Africans, mainly Zulu, Swazi and Ndebele, and the mean gestational age was 6.5 months (SD 1.6). The inclusion criteria for participation in the study were that the participant should have attended at least one antenatal care clinic during the current pregnancy, and that she was 18 years of age or older; there was no gestational age limit. Antenatal women were recruited systematically (every consecutive patient visiting the clinic) from 63 of 74 primary care clinics and community health centres in all the 6 sub-districts of the Nkangala District; 11 clinics were not included since four refused and in seven clinics the field workers could not be recruited or resigned before data collection. The target was to interview at least 30 pregnant women per study clinic. Approval for the study was obtained from the Human Sciences Research Council ethics committee and health authorities at the provincial, district, sub-district and clinic levels as well as the CDC Center for Global Health (FWA 6347).

Data were collected from April to June 2010 at primary health care facilities in Nkangala District. Women who were 18 years and older and had come for their second antenatal care visit were eligible to participate in the study. A team of trained research assistants visited the facilities daily to conduct the interviews until the sample was reached. The interviews were conducted in Zulu, Swati and Ndebele (main spoken languages, which are also well understood by other ethnic groups) and took 45 minutes to complete. Written informed consent was obtained from all the participants. The research assistants provided an explanation of the nature and purpose of the study, including assurance of privacy and confidentiality. The interviews were conducted in a private room at the healthcare facility.

### Measures

Participants provided data on their socio-demographics, including obstetric indicators (parity, current pregnancy planned, unhappy about current pregnancy), HIV status, sexual behaviour, history of physical partner abuse, alcohol use and a measure of psychological distress (anxiety and depression). Alcohol use was assessed by the frequency of past month use of alcohol. To determine abuse history, participants were asked ‘Have you experienced physical abuse by your partner/husband in the past 6 months?’ (physical abuse included being hit, slapped, kicked, bit, pushed, shoved or physically hurt in another way by the partner).

In addition, participants completed the The Kessler Psychological Distress Scale (K-10), which is a measure of global psychological distress on perceived frequency of occurrence of significant psychiatric related symptoms over the preceding 30 days [19,20]. There was significant agreement among HIV patients between the K-10 and the MINI-defined depressive and anxiety disorders, with the best screening cut-off score of 28 [21]. A receiver operating characteristic (ROC) curve analysis indicated that the K-10 showed agreeable sensitivity and specificity in detecting depression (area under the ROC curve, 0.77), generalized anxiety disorder (0.78), and posttraumatic stress disorder (PTSD) (0.77), with the best cut off of 28 [22]. The internal reliability coefficient for the K-10 in this study was alpha = 0.89. Knowledge of HIV transmission was assessed with 4 items, e.g., “Can an HIV-infected mother infect her baby with HIV during pregnancy”). Cronbach alpha for the four items in HIV transmission knowledge was .68 for this sample.

### Data analysis

The IBM statistical software Statistical Package for Social Sciences (SPSS version 19.0 for Windows; Chicago, IL, USA) was used for data analyses. Descriptive statistics were used to describe the sample. Bivariate analysis and forced multiple logistic regressions were used to investigate associations between the predictor variables and the outcomes: 1) Two or more sexual partners in the past 12 months, 2) Never used condom with primary partner in the past 3 months, 3) History of sexually transmitted infection (STI) (other than HIV) in the past 12 months, and 4) HIV positive or unknown HIV status of a sexual partner. The factors that were found to be significant in the bivariate analysis were included in the full model. Associations were considered significant at p < 0.05.

## Results

### Sample characteristics and sexual HIV risk behaviour

The age of the study participants was on average 26.6 years (SD = 6.1), ranging from 18 to 47 years. Most participants (91%) had Grade 8 or more education and 70% were single. For more than half of the participants (55%), the current pregnancy had not been planned and they had on average 1 child (SD = 1.2). For those who reported to have been tested for HIV, 19.8% were HIV positive, 16% believed that antiretrovirals (ARVs) can cure HIV and 22% believed that someone on antiretroviral treatment cannot transmit the HIV virus. In terms of psychosocial distress, 8.5% reported physical partner violence in the past 6 months, 17.8% psychological distress and 6.5% any alcohol use in the past month. The majority (63%) had never used a condom with their primary sexual partner in the past 3 months, 60% were not aware of the HIV status of their sexual partner, 7.6% had a casual sexual partner in the past 3 months, 20% had two or more sexual partners in the past 12 months and 17.3% reported to have been diagnosed with a sexually transmitted infection (STI) (other than HIV) in the past 12 months (see Table 1).

**Table 1:** Sample characteristics and sexual HIV risk behaviour of pregnant women (N = 1502)

**Variables**	**n or M**	**% or SD**
**Socio-economic factors**		
Age (years), M (s.d.)	26.6	6.1
*Education (years or Grades)*		
Grade 7 or less, n (%)	134	9.0
Grade 8–11, n (%)	698	46.6
Grade 12 or more, n (%)	665	44.4
*Marital status*		
Single, n (%)	1025	69.8
Married or cohabitating, n (%)	444	30.2
**Obstetrics**		
Number of own children, M (s.d)	1.2	1.2
Number of antenatal care visits, M (s.d.)	3.4	1.5
Current pregnancy not planned, n (%)	824	55.2
Father accompany to antenatal clinic, n (%)	435	29.4
**HIV related variables**		
HIV knowledge scores, M (s.d.)	2.5	1.1
Belief ARVs can cure HIV, n (%)	231	16.3
Belief in someone treated with ARVs cannot transmit the HIV virus, n (%)	318	22.4
*HIV status*		
HIV positive, n (%)	278	19.8
HIV negative, n (%)	1123	80.2
HIV status unknown, n (%)	101	[6.7]
**Psychosocial distress and alcohol use**		
Past month (any) alcohol use, n (%)	93	6.5
Physical partner violence in the past 6 months, n (%)	123	8.5
Psychological distress (Kessler 10 ≥28 scores), n (%)	267	17.8
**Sexual HIV risk factors**		
Two or more sexual partners in the past 12 months, n (%)	155	19.9
Casual sexual partner in the past 3 months, n (%)	107	7.6
History of STI (other than HIV) in the past 12 months, n (%)	254	17.3
*Condom use with primary partner in the past 3 months*		
Never, n (%)	899	63.3
Less than half of the time, n (%)	114	8.0
Half of the time, n (%)	81	5.7
More than half of the time, n (%)	99	7.0
Every time, n (%)	228	16.0
*Knows partner’s HIV status*		
HIV positive, n (%)	77	12.6
HIV negative, n (%)	532	87.4
Do not know/refused to answer, n (%)	893	[59.5]

N = number;% = percent; M = Mean; SD/s.d. = Standard deviation.

### Predictors of sexual HIV risk behaviours

In multivariate analysis being single (OR = 1.62; CI = 1.00-2.62) and alcohol use in the past 12 months (OR = 4.38; CI = 2.63-7.31) were found to be associated with having had two or more sexual partners in the past 12 months. Pregnant women with fewer antenatal visits (OR = 0.91; CI = 0.85-0.99), those who reported being HIV negative (OR = 0.39; CI = 0.29-0.52), and those who had not used alcohol in the past month (OR = 0.55; CI = 0.35-0.86) were more likely to have never used condoms with their primary partner in the past three months (see Table 2).

**Table 2:** Results of regression analyses predicting multiple sexual partners and never having used condoms among pregnant women

	**Two or more sexual partners in past 12 months**		**Never condom use with primary partner in past 3 month**	
	**UOR (95% CI)**	**AOR**^**1 **^**(95% CI)**	**UOR (95% CI)**	**AOR**^**2 **^**(95% CI)**
**Socio-economic factors**				
Age (years)	0.96 (0.93-0.98)**	0.98 (0.94-1.02)	1.00 (0.99-1.02)	---
Education (years)	0.92 (0.84-1.01)	---	0.96 (0.90-1.03)	---
Single (Base = married)	2.05 (1.34-3.13)***	1.62 (1.00-2.62)*	0.80 (0.63-1.02)	---
**Obstetrics**				
Number of own children	0.82 (0.71-0.96)**	0.91 (0.74-1.13)	1.01 (0.93-1.10)	---
Number of antenatal care visits	0.93 (0.83-1.05)	---	0.90 (0.84-0.96)**	0.91 (0.85-0.99)*
Current pregnancy not planned (Base = planned)	1.27 (0.91-1.78)	---	1.04 (0.84-1.29)	---
Father accompany to antenatal clinic (Base = No)	1.25 (0.88-1.78)	---	0.79 (0.63-0.99)*	0.83 (0.64-1.06)
**HIV related variables**				
HIV knowledge scores	1.04 (0.89-1.20)	---	0.94 (0.85-1.04)	---
Belief ARVs can cure HIV (Base = No)	0.97 (0.49-1.27)	---	0.97 (0.72-1.30)	---
Belief in someone treated with ARVs cannot transmit the HIV virus (Base = No)	0.97 (0.65-1.45)	---	1.00 (0.77-1.30)	---
HIV positive (Base = HIV negative)	1.11 (0.73-1.68)	---	0.44 (0.33-0.57)***	0.39 (0.29-0.52)***
**Psychosocial distress and alcohol use**				
Past month (any) alcohol use (Base = No)	4.77 (2.99-7.63)***	4.38 (2.63-7.31)***	0.56 (0.37-0.86)**	0.55 (0.35-0.86)**
Physical partner violence in the past 6 months (Base = No)	1.81 (1.09-2.98)*	1.59 (0.90-2.80)	0.83 (0.57-1.22)	---
Psychological distress (Kessler 10 ≥ 28 scores) (Base = <28 scores)	1.64 (1.11-2.41)	---	1.02 (0.77-1.34)	---

^1^Hosmer-Lemeshow χ^2^ = 9.24, *p* = 0 .323; Nagelkerke R^2^ = 0.08; ^2^Hosmer-Lemeshow χ^2^ = 8.95, *p* = 0 .177; Nagelkerke R^2^ = 0.06; UOR = Unadjusted Odds Ratio; AOR = Adjusted Odds Ratio; CI = Confidence Interval.

Further, in multivariate analysis being HIV positive (OR = 1.94; CI = 1.39-2.72), having experienced physical partner violence in the past six months (OR = 2.08; CI = 1.32-3.30), and those who had psychological distress (OR = 1.84; CI = 1.29-2.62) were found to be associated with having been diagnosed with an STI (other than HIV) in the past 12 months. Pregnant women with lower education (OR = 0.91; CI = 0.84-0.99), those whose current pregnancy had not been planned (OR = 1.73; CI = 1.36-2.20), those with non-antenatal care attendance by expectant father (OR = 0.53; CI = 0.41-0.69), those who believed that ARVs can cure HIV (OR = 1.41; CI = 1.00-1.97), and those who reported to be HIV positive (OR = 5.01; CI = 3.42-7.34) were more likely to have a partner with HIV positive or HIV unknown status (see Table 3).

**Table 3:** Results of regression analyses predicting history of STI and HIV positive or unknown status of sexual partner

	**History of STI (other than HIV) in the past 12 months**		**HIV status of partner is positive or unknown**	
	**UOR (95% CI)**	**AOR**^**1 **^**(95% CI)**	**UOR (95% CI)**	**AOR**^**2 **^**(95% CI)**
**Socio-economic factors**				
Age (years)	1.12 (1.00-1.04)	---	1.00 (0.98-1.02)	---
Education (years)	0.93 (0.86-1.01)	---	0.88 (0.82-0.94)***	0.91 0.84-0.99)*
Single (Base = married)	1.04 (0.77-1.40)	---	1.18 (0.93-1.48)	---
**Obstetrics**				
Number of own children	1.18 (1.03-1.34)*	1.10 (0.99-1.24)	1.07 (0.96-1.19)	---
Number of antenatal care visits	0.97 (0.88-1.06)	---	1.02 (0.95-1.09)	---
Current pregnancy not planned (Base = planned)	0.95 (0.73-1.25)	---	1.75 (1.41-2.17)***	1.73 (1.36-2.20)***
Father accompany to antenatal clinic (Base = No)	1.11 (0.83-1.49)	---	0.56 (0.44-0.70)***	0.53 (0.41-0.69)***
**HIV related variables**				
HIV knowledge scores	1.12 (0.98-1.27)	---	0.94 (0.85-1.04)	---
Belief ARVs can cure HIV (Base = No)	0.90 (0.64-1.27)	---	1.51 (1.11-2.05)**	1.41 (1.00-1.97)*
Belief in someone treated with ARVs cannot transmit the HIV virus (Base = No)	1.58 (1.12-2.23)**	1.24 (0.97-2.11)	0.96 (0.74-1.25)	---
HIV positive (Base = HIV negative)	2.13 (1.56-2.91)***	1.94 (1.39-2.72)***	5.17 (3.57-7.48)***	5.01 (3.42-7.34)***
**Psychosocial distress and alcohol use**				
Past month (any) alcohol use (Base = No)	1.29 (0.75-2.20)	---	1.33 (0.84-2.09	---
Physical partner violence in the past 6 months (Base = No)	2.90 (1.94-4.34)***	2.08 (1.32-3.30)***	1.07 (0.73-1.57)	---
Psychological distress (Kessler 10 ≥ 28 scores) (Base = <28 scores)	2.19 (1.60-2.99)**	1.84 (1.29-2.62)***	1.17 (0.88-1.54)	---

^1^Hosmer-Lemeshow χ^2^ = 7.97, *p* = 0 .335; Nagelkerke R^2^ = 0.07; ^2^Hosmer-Lemeshow χ^2^ = 7.49, *p* = 0 .485; Nagelkerke R^2^ = 0.15; UOR = Crude Odds Ratio; AOR = Adjusted Odds Ratio; CI = Confidence Interval.

## Discussion

This study sought to assess sexual HIV risk behaviour and its associated factors among pregnant women in Mpumalanga, South Africa. Results indicate that among pregnant women (6.5 months mean gestational age) HIV sexual risk behaviour was quite prevalent, including unprotected sex, multiple partners and sexual partners of unknown serostatus. Regarding sexual risk, previous studies had similar findings related to increased HIV risk behaviour (unprotected sex, multiple sexual partners) among pregnant women [10,11,23-25].

Further, the study found that being single and alcohol use were associated with multiple sexual partners. Other studies in South Africa also found that drinking prior to pregnancy recognition or during pregnancy and being single was associated with having a greater number of sexual partners or a greater history of sexual risk-taking [26,27]. Moreover, fewer antenatal visits, being HIV negative and not having used alcohol were associated with unprotected intercourse. This seems to show the importance of antenatal care attendance, which can be used to reinforce condom use. Also other studies [28,29] show that being HIV positive was associated with protected sexual intercourse. Previous studies also found that alcohol use was inconsistently related to protective behaviours (e.g., condom use) [30].

This study found, as in other studies [31,32], that being HIV positive was associated with having been diagnosed with a sexually transmitted infection (other than HIV). Further, having experienced physical partner violence and psychological distress were found to be associated with having been diagnosed with an STI (other than HIV). This finding is conforming to other studies about the co-occurrence of intimate partner violence and STIs (including HIV) [15,16,33-35] and psychological distress has been found to be associated with HIV risk behaviour [36,37]. In a study among pregnant women in rural Haiti, results showed that gender and power factors were most significant for condom use. These results suggest the need to create prevention interventions that restore power imbalances, strengthen communication skills [18] and partner communication on sexual matters [38]. Treating intimate partner violence, mental health and alcohol use problems may aid in reducing HIV infection [36].

Finally, educational factors (lower education, the belief that antiretrovirals can cure HIV, unplanned pregnancy), lack of male involvement (non-antenatal care attendance by expectant father) and being HIV positive were found to be associated with having a partner with HIV positive or unknown status. Having unprotected sexual intercourse with partners of HIV positive or unknown HIV status includes an increased HIV risk and should be avoided and calls for improved partner communication on sexual matters [38]. In addition, health education should address misconceptions about the effects of antiretrovirals. In this study HIV knowledge was not found to be associated with HIV risk behaviour, unlike in a previous study in South Africa [39].

### Study limitations

The measures used in the study were all by self report, so there is a possibility of a degree of biased reporting. It is possible that respondents underreported sexual risk behaviour. Furthermore, this study was based on data collected in a cross-sectional survey. We cannot, therefore, ascribe causality to any of the associated factors in the study. Prospective studies are required to confirm the sexual behaviour findings.

## Conclusion

This study identified high levels of HIV risk behaviour among women during pregnancy in South Africa. Multivariate analysis revealed significant factors (being single, lower education, the belief that antiretrovirals can cure HIV, fewer antenatal care visits, unplanned pregnancy, non-antenatal care attendance by expectant father, HIV status, alcohol use, physical partner violence and psychological distress) associated with various sexual HIV risk behaviour among pregnant women. Recent studies have highlighted the heightened risk of HIV transmission during pregnancy. This increased risk of HIV transmission and the burst of viral particles in the blood (viremia) associated with HIV infection make unprotected sex during pregnancy especially dangerous to mothers. The results of this study call for the need of targeted HIV risk reduction interventions for pregnant women [40].

## Competing interests

The authors declare that they have no competing interests.

## Authors’ contributions

KP was the main contributor to the conceptualization of the study. KP and GM contributed significantly to the first draft of the paper and all authors contributed to the subsequent drafts and finalization. Both authors read and approved the final manuscript.

## Pre-publication history

The pre-publication history for this paper can be accessed here:

http://www.biomedcentral.com/1471-2393/13/57/prepub
